# MicroRNA Mimics Based on the miR-15/107 Consensus Sequence Sensitise NSCLC Cells to Targeted Therapy

**DOI:** 10.3390/ijms27062701

**Published:** 2026-03-16

**Authors:** Carien Carpenter, Nina Simmons, William J. H. Davis, Madeleine Thompson, Nico van Zandwijk, Catherine J. Drummond, Glen Reid

**Affiliations:** 1 Department of Pathology and Molecular Medicine, University of Otago, Dunedin 9054, New Zealand; carca489@student.otago.ac.nz (C.C.); cath.drummond@otago.ac.nz (C.J.D.); 2 Maurice Wilkins Centre for Molecular Biodiscovery, The University of Auckland, Auckland 1142, New Zealand; 3 Sydney Local Health District, Sydney, NSW 2065, Australia; nico.vanzandwijk@sydney.edu.au; 4 Faculty of Medicine and Health, The University of Sydney, Sydney, NSW 2050, Australia

**Keywords:** microRNA, targeted therapy, drug tolerance, lung adenocarcinoma, EGFR, KRAS

## Abstract

Non-small cell lung cancer (NSCLC) is the leading cause of lung cancer deaths, with resistance to targeted therapies posing a major clinical challenge. Drug-tolerant persister (DTP) cells are key contributors to resistance, and targeting them offers new strategies to enhance existing treatments. MicroRNAs (miRNAs), particularly the tumour-suppressive miR-15/107 family, offer promise due to their ability to target multiple oncogenic pathways. This study evaluated a synthetic consensus miRNA mimic, conmiR-15/107, in NSCLC cell line models. Dose–response assays showed robust, dose-dependent growth inhibition in both EGFR-mutant (PC9) and KRAS-mutant (H358 and A549) lung adenocarcinoma cells, but not in the human bronchial epithelial cell line BEAS-2B. When combined with EGFR inhibitors (osimertinib and gefitinib) in PC9 cells, the mimics showed a higher rate of growth inhibition compared with the controls and reduced IC_50_ values. Similarly, conmiR-15/107 enhanced growth inhibition by the KRAS inhibitors sotorasib and adagrasib in H358 cells. RT-qPCR confirmed downregulation of conmiR-15/107 targets, including MEK1, BCL2 and BRCA1, suggesting a multi-target mechanism of action. Long-term assays showed that the mimics reduced the survival and delayed the proliferation of DTPs in osimertinib-treated PC9 cells as well as sotorasib-treated H358 cells. These findings support conmiR-15/107 as a potential adjunct to targeted therapy, capable of enhancing treatment efficacy and delaying resistance in lung adenocarcinoma.

## 1. Introduction

Lung cancer is the predominant cause of cancer-associated mortality globally, highlighting the critical demand for novel and more durable therapeutic approaches [1]. Although targeted therapies have significantly improved outcomes in non-small cell lung cancer (NSCLC), their long-term efficacy is often hindered by the inevitable development of drug resistance [2]. Epidermal growth factor receptor (EGFR) tyrosine kinase inhibitors (TKIs), such as the first-generation gefitinib and the third-generation agent osimertinib, have improved progression-free survival when compared with chemotherapy in patients with EGFR-mutant NSCLC [3,4]. Despite frequent responses, resistance inevitably emerges through mechanisms such as secondary mutations in EGFR (e.g., T790M, C797S), activation of bypass signalling pathways (e.g., MET or HER2 amplification) or phenotypic changes such as transdifferentiation into small cell lung cancer (SCLC) or epithelial-to-mesenchymal transition (EMT) [5]. Similarly, the recent discovery and clinical development of KRAS^G12C^ inhibitors, such as sotorasib and adagrasib [6,7,8,9], have provided a novel approach for this previously “undruggable” target, but adaptive resistance limits the durability of responses for both of these new drugs [10,11].

A growing body of evidence indicates that drug-tolerant persister (DTP) cells play a central role in the emergence of resistance to targeted therapies [12,13,14]. These rare, slow-cycling subpopulations survive initial drug exposure through reversible, non-genetic adaptations, such as chromatin remodelling [15], altered metabolic states [16] or activation of survival pathways [11,17]. Although DTPs typically represent only a small fraction of the tumour, they can persist under prolonged therapeutic pressure, eventually begin to proliferate (known as drug-tolerant expanded persisters, DTEPs) and give rise to genetically resistant clones [18,19,20], posing a major challenge to the long-term efficacy of EGFR and KRAS inhibitors [11,17]. The lack of mutational alterations in DTPs makes them difficult to target, and while vulnerabilities in several pathways altered in DTPs have been explored, to date, none have been broadly effective (reviewed in [12,13]).

MicroRNAs (miRNAs) may be particularly well suited to the challenge of targeting DTPs due to their ability to post-transcriptionally regulate multiple genes and pathways simultaneously [21]. MiRNAs are short (17–24 nucleotide) non-coding RNAs that associate with the RNA-induced silencing complex (RISC) and bind complementary recognition sequences in target mRNAs to inhibit their translation [22]. Among tumour-suppressive miRNAs, the miR-15/107 family has garnered particular interest due to its widespread dysregulation across cancer types, including NSCLC, and its involvement in resistance-related pathways [23]. Members of this family play key roles in regulating key oncogenic processes, including apoptosis, cell proliferation, cell cycle progression and EMT [24]. Targets of this family include MEK1, MAPK3 and CRAF, which are all components of MAPK signalling [25,26]. Notably, many of these targets are downstream of receptor tyrosine kinase signalling pathways, including EGFR, suggesting that miR-15/107-based therapies may offer benefit when combined with EGFR inhibitors.

Multiple preclinical investigations have examined the restoration of miRNA expression using synthetic mimics as a therapeutic approach to improve the effectiveness of existing targeted therapies [27]. In preclinical models, restoration of miR-34a using a mimic has been reported to re-sensitise cancer cells to EGFR inhibitors, including erlotinib [28] and osimertinib [29]. In KRAS-mutant NSCLC cell lines, Fanini et al. reported that miR-16 mimic transfection re-sensitised cells to EGFR inhibition with erlotinib and surpassed the therapeutic effect of dual EGFR/MEK blockade using selumetinib–erlotinib [25]. Our lab developed a series of synthetic consensus sequence mimics, “conmiR-15/107,” based on members of the highly conserved miR-15/107 superfamily [30], most of which share the 5′-AGCAGC-3′ seed sequence [23]. These engineered constructs were designed to replicate the tumour-suppressive functions of the entire family by targeting oncogenic effectors from multiple pathways. In vitro, conmiR-15/107 constructs induced robust growth inhibition across several cancer cell lines, attributed to downregulation of genes such as BCL2 and CCND1 [30]. In vivo studies in xenograft models of PM and NSCLC demonstrated significant tumour growth suppression, further supporting its therapeutic potential.

Therapeutic applications of miRNA mimics and inhibitors are increasingly being explored, with several candidates advancing to clinical trials. For instance, MRX34, a liposomal miR-34a mimic, was among the first to be tested clinically in advanced solid tumours, where it showed early signs of antitumour efficacy; however, the study was prematurely terminated as a result of severe immune-mediated toxicities [31]. Following this, the phase I TARGOmiR study assessed the delivery of a miR-16 mimic via EGFR-targeted minicells (EnGeneIC Dream Vectors) in pleural mesothelioma (PM) patients, providing evidence of feasibility and potential clinical benefit [32]. This trial was open to NSCLC patients, but none were enrolled, and thus, this avenue of investigation remains unexplored.

Based on the known targets of members of the miR-15/107 family, the goal of this study was to investigate the therapeutic potential of the conmiR-15/107 consensus mimics in oncogene-driven NSCLC models. Specifically, we aimed to evaluate the ability of these constructs to enhance the efficacy of targeted therapies and inhibit DTPs.

## 2. Results

### 2.1. conmiR-15/107 Shows Dose-Dependent Inhibitory Effect on Cell Proliferation

**Figure 1:**
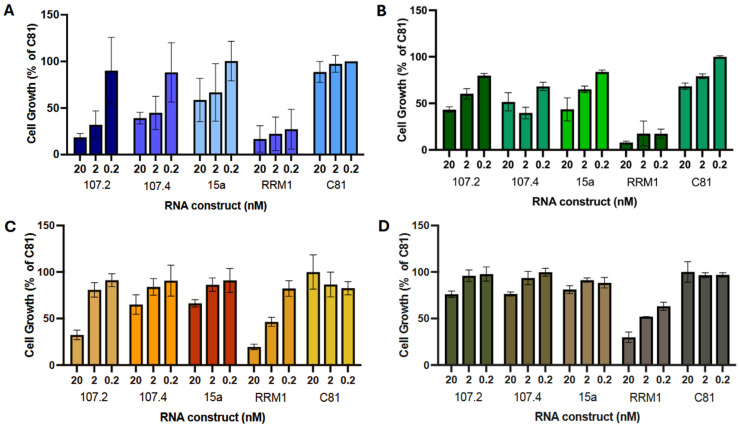
Inhibition of cell proliferation by miRNA constructs in lung cancer cell lines. PC9 (**A**), H358 (**B**), A549 (**C**) and BEAS2B (**D**) cells were transfected with miRNA constructs conmiR-15/107.2, conmiR-15/107.4 or miR-15a. C81 (negative control) and RRM1 (positive control) were included as transfection controls. Cell proliferation after 96 h was measured in triplicate across a range of construct concentrations and expressed as a percentage relative to C81-transfected cells (normalised to 100%). Data shown are representative of three independent biological replicates. Error bars indicate mean ± SD of technical repeats.

In a previous study, we showed that the conmiR-15/107 constructs have an inhibitory effect on proliferation in multiple cancer types, including NSCLC cells [30]. Here, the effects of conmiR-15/107 constructs on cell proliferation were tested in the oncogene-driven adenocarcinoma cell lines PC9 (EGFR exon 19 deletion), H358 (KRAS^G12C^) and A549 (KRAS^G12S^), and compared with effects on the BEAS-2B cell line as a normal lung epithelial control. In PC9 cells, conmiR-15/107.2 and conmiR-15/107.4 induced a dose-dependent inhibition of proliferation, with approximately 50% inhibition at the highest concentration (Figure 1A). A miR-15a mimic showed a similar, though slightly less potent, effect. In H358 cells, both constructs again caused a dose-dependent inhibition of proliferation, with a maximum of approximately 60% inhibition at 20 nM, with similar results observed with miR-15a (Figure 1B). In A549 (Figure 1C), conmiR-15/107.2 induced 70% inhibition and conmiR-15/107.4 40% inhibition at 20 nM. In BEAS-2B cells, miR-15a, conmiR-15/107.2 and conmiR-15/107.4 had a lesser effect on cell proliferation, except at the highest concentration (20 nM), where they had a 25% inhibition (Figure 1D).

### 2.2. miR-15/107 Sensitises Lung Adenocarcinoma Cells to Targeted Therapy

Individual members of the miR-15/107 family have been shown to sensitise NSCLC to targeted therapy [25], and previously we showed that the conmiR-15/107 constructs could sensitise PM and NSCLC cells to gemcitabine treatment [30]. To investigate whether conmiR-15/107 constructs sensitised EGFR- and KRAS^G12C^-mutant lung adenocarcinoma cells to targeted therapy, PC9 and H358 cells were transfected with 2 nM of conmiR-15/107.2, conmiR-15/107.4 or control, followed by treatment with increasing concentrations of the EGFR inhibitors osimertinib and gefitinib, or the KRAS inhibitors sotorasib and adagrasib.

**Figure 2:**
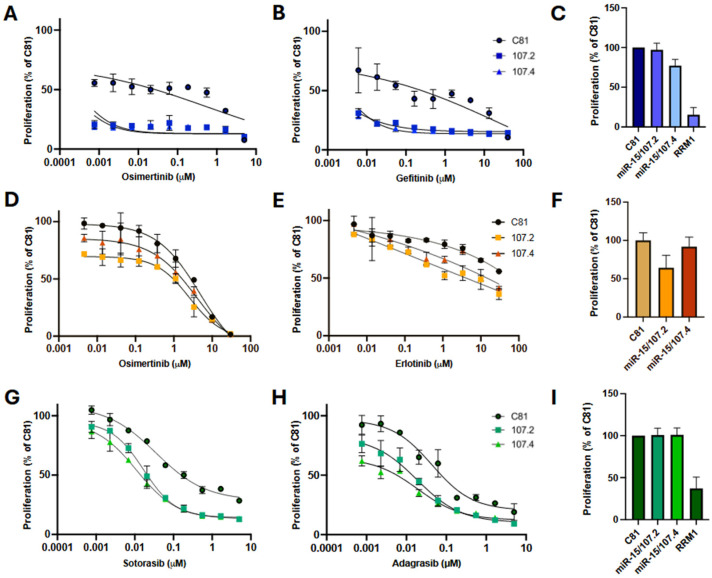
conmiR-15/107 constructs increase targeted therapy efficacy. Dose–response curve showing proliferation of PC9 (**A**,**B**), A549 (**D**,**E**) and H358 (**G**,**H**) cells reverse-transfected with C81 (negative control), conmiR-15/107.2 or conmiR-15/107.4 (each at a final concentration of 2 nM), and 24 h later treated with increasing concentrations of osimertinib (**A**,**D**), gefitinib (**B**), erlotinib (**E**), sotorasib (**G**) and adagrasib (**H**). Proliferation was measured after 96 h of drug treatment. Estimated IC_50_ values (*n* = 3) for sotorasib: 36 ± 4 nM for control versus 14 ± 3 (*p* < 0.05) and 12 ± 4 nM (*p* < 0.05) for conmiR-15/107.2 and conmiR-15/107.4, respectively; for adagrasib: 42 ± 1 versus 17 ± 3 (*p* < 0.05) and 12 ± 5 nM (*p* < 0.01). Data shown are representative of three independent biological replicates. Error bars indicate mean ± SD of technical repeats normalised to C81 control. (**C**,**F**,**I**) Relative proliferation of mimic-transfected cells in PC9 (**C**), H358 (**F**) and A549 (**I**) cells, normalised to C81 controls. Data represent mean ± SD of three biological replicates. In panels (**A**,**B**,**G**,**H**), all values significantly different (*p* < 0.05, paired *t*-test) between C81 control and each consensus mimic, except for the respective highest (**A**,**B**) or lowest (**G**,**H**) concentration.

PC9 cells transfected with conmiR-15/107.2 and conmiR-15/107.4 were significantly more sensitive to both osimertinib and gefitinib treatment at all concentrations tested, with all combinations leading to >50% inhibition of proliferation (Figure 2A,B), precluding comparison of IC_50_ values, but suggestive of a synergistic interaction (Supplementary Figure S1). Both conmiR-15/107.2 and conmiR-15/107.4 also sensitised PC9 cells to erlotinib, although to a lesser extent (Supplementary Figure S2). Control experiments confirmed that 2 nM mimic alone had minimal impact on cell viability and that transfection efficiency was consistent across conditions (Figure 2C,F,I). To determine whether conmiR-15/107.2 and conmiR-15/107.4 could increase sensitivity to EGFR inhibitors in EGFR wild-type cells, we transfected A549 cells with the constructs and treated them with osimertinib and gefitinib. As expected, A549 cells were considerably less sensitive than PC9 cells to osimertinib (Figure 2D) and essentially insensitive to erlotinib (Figure 2E). Although the combination with conmiR-15/107.2 and conmiR-15/107.4 increased sensitivity to both drugs, these changes were not statistically significant. The mimics also failed to significantly increase sensitivity to the MEK inhibitor trametinib (Supplementary Figure S2).

Next we evaluated whether conmiR-15/107 constructs sensitise KRAS-mutant NSCLC cells to KRAS^G12C^ inhibitors. H358 cells were transfected with 2 nM of conmiR-15/107.2, conmiR-15/107.4 or control, followed by treatment with increasing concentrations of sotorasib or adagrasib. Both constructs enhanced drug sensitivity to KRAS inhibitors, with reduced cell viability (Figure 2G,H) and lowered estimated IC_50_ values, again suggesting a synergistic interaction (Supplementary Figure S1). H358 cells transfected with conmiR-15/107.2 and conmiR-15/107.4 were significantly more sensitive to both sotorasib and adagrasib treatment at all concentrations of targeted therapy tested.

### 2.3. miR-15/107 Consensus Mimics Regulate Multiple Targets in PC9 Cells

**Figure 3:**
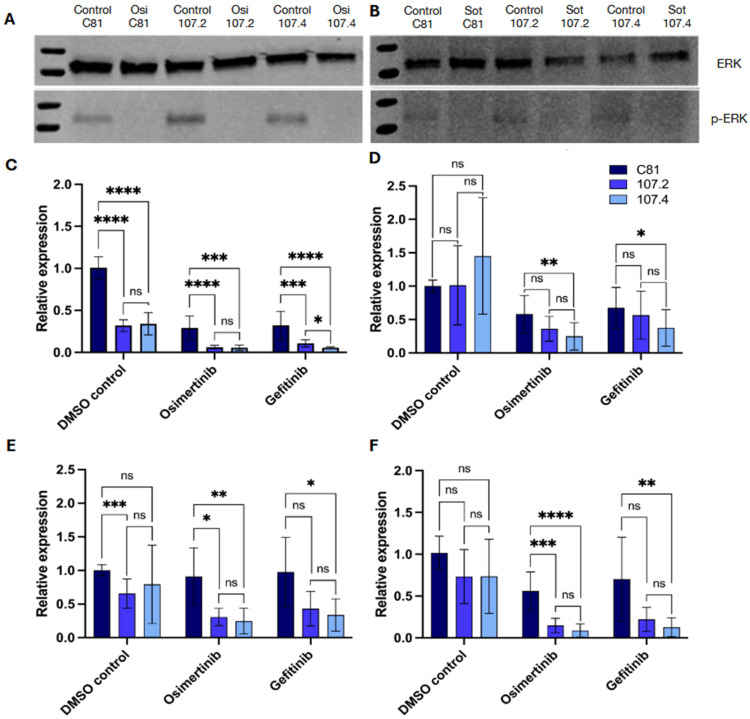
Target mRNA and protein expression in PC9 and H358 cells following transfection and drug treatment. PC9 (**A**) and H358 (**B**) cells were transfected with either C81, conmiR-15/107.2 or conmiR-15/107.4 (final concentration 2 nM) and 72 h later treated with osimertinib (**A**) or sotorasib (**B**) for 4 h before protein isolation**.** Western blots were performed to determine protein expression of ERK and p-ERK. (**C**–**F**) PC9 cells were reverse-transfected with C81, conmiR-15/107.2 or conmiR-15/107.4 and 24 h later treated with DMSO, osimertinib (2 μM) or gefitinib (5 μM). After 48 h of drug treatment, RNA was isolated, and RT-qPCR was used to measure mRNA expression of (**C**) BCL2, (**D**) CCND1, (**E**) MEK1 and (**F**) BRCA1. Data represent the mean ± SD of three biological replicates (*n* = 3). *p* < 0.05 (*), *p* < 0.01 (**), *p* < 0.001 (***), *p* < 0.0001 (****); ns = not significant (unpaired, non-parametric Mann–Whitney test).

Given the clear inhibitory effect of both consensus mimics on cell proliferation, we next investigated possible mechanistic effectors of the constructs. To investigate the regulatory impact of conmiR-15/107 constructs on known miR-15/107 family targets, PC9 cells were transfected with 2 nM of C81, conmiR-15/107.2 or conmiR-15/107.4 and treated with DMSO, 2 μM osimertinib or 5 μM gefitinib. Western blot results indicated no reduction in phosphorylated ERK following transfection with conmiR-15/107.2 or conmiR-15/107.4 in PC9 and H358 cells (Figure 3A,B); therefore, subsequent experiments focused on identifying alternative targets. As both BCL2 and CCND1 were confirmed as targets of the conmiR-15/107 constructs in PM cell lines [30], we investigated whether this was also the case in NSCLC models. BCL2, an anti-apoptotic gene, was consistently downregulated across all treatment conditions (Figure 3C). In DMSO-treated cells, BCL2 expression was reduced ~3-fold in both conmiR-transfected conditions compared with C81. In osimertinib-treated cells, reductions reached ~5-fold, while gefitinib treatment resulted in ~3-fold (conmiR-15/107.2) and ~6-fold (conmiR-15/107.4) downregulation. In contrast, neither conmiR-15/107.2 nor conmiR-15/107.4 significantly altered CCND1 mRNA levels under any condition (Figure 3D).

As MEK1, a component of the MAPK/ERK pathway and involved in EGFR signalling, has been shown to be downregulated by miR-15/107 family members [25], we investigated whether the consensus mimic constructs affected its expression. Following transfection with both constructs, MEK1 was found to be significantly downregulated under all conditions (Figure 3E). In DMSO-treated cells, MEK1 expression decreased ~1.4-fold. Under osimertinib, MEK1 was reduced ~3-fold in both construct conditions. Gefitinib treatment led to ~2-fold (conmiR-15/107.2) and ~3-fold (conmiR-15/107.4) downregulation. Finally, BRCA1, involved in DNA damage repair and another potential target [33], was strongly suppressed following conmiR-15/107 transfection under drug treatment (Figure 3F). Under osimertinib, BRCA1 expression was reduced ~3.7-fold (107.2) and ~6-fold (107.4). Under gefitinib treatment, BRCA1 decreased ~3-fold (107.2, non-significant) and ~5.5-fold (107.4) relative to control.

### 2.4. miR-15/107 Inhibits the Survival of DTPs

**Figure 4:**
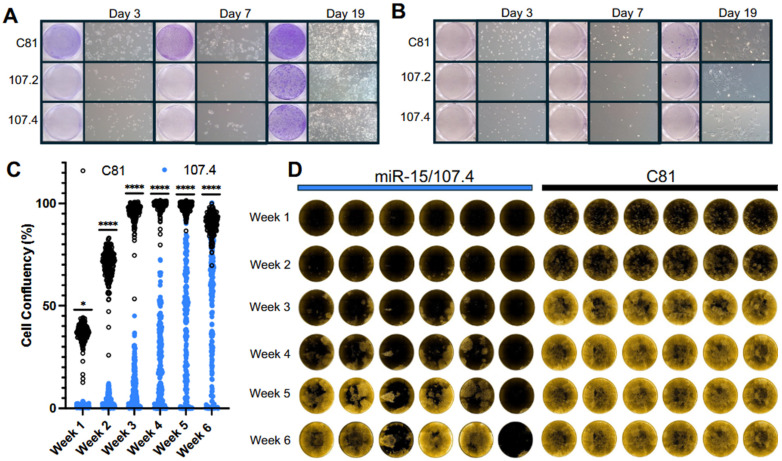
Effect of consensus mimics on DTP survival and proliferation following long-term drug treatment. PC9 and H358 cells were reverse-transfected with control (C81) or miR-15/107.4 constructs (2 nM) and further treated with targeted therapy for the indicated times. Crystal violet staining and phase-contrast images of PC9 (**A**) and H358 (**B**) cells were generated at day 3, day 7 and day 19 of treatment. For each time point: scanned images of 6-well plates stained with crystal violet, showing osimertinib (**A**) or sotorasib (**B**) treated cells transfected with C81, conmiR-107.2 or conmiR-107.4 constructs are shown, with corresponding phase-contrast microscopy images (200 µm scale) depicting representative proliferation of transfected cells in each well. The experiment was independently repeated three times with consistent results (*n* = 3). (**C**) mCherry-transduced PC9 cells were reverse-transfected with miR-15.107.4 or C81 in glass-bottom 96-well plates and 24 h later treated with osimertinib. Plates were imaged weekly using the Opera Phenix High Content Screening System (see Section 4.6). The dot plot shows estimated cell confluency (%) per well across multiple time points, derived from Phenix imaging software (Signal Image Artist (Version 1)). Data represent three plates (180 wells) per condition, with Mann–Whitney tests performed at each time point between C81 and 107.4-transfected conditions. (**D**) Fluorescent imaging of proliferation of mCherry-transduced PC9 cells following transfection with miR-15.107.4 or C81 and subsequent treatment with osimertinib for 6 weeks. Images of representative wells from three independent plates per condition are shown. Each row corresponds to a different time point, with images using the Phenix high-content imaging system taken weekly to monitor proliferation and persistence (see Supplementary Figure S3 for images from additional wells and Supplementary Table S3 for all confluency data). *p* < 0.05 (*), *p* < 0.0001 (****).

As conmiR-15/107 transfection enhanced the sensitivity of PC9 cells to osimertinib and H358 to sotorasib, we next assessed the impact of these constructs on the survival of DTPs and eventual appearance of DTEPs during extended drug treatment. PC9 cells are an established model of drug tolerance [15,18,34], and we recently showed that H358 cells also exhibit transcriptional features consistent with this state [35]. PC9 cells were transfected with conmiR-15/107.2, conmiR-15/107.4 or control mimics (2 nM) and then treated with osimertinib (2 µM) or DMSO. Cell survival was monitored over 19 days, and it was found that the constructs greatly reduced the number of DTPs in both osimertinib-treated PC9 cells (Figure 4A) and sotorasib-treated H358 cells (Figure 4B). The effect was more pronounced in osimertinib-treated PC9 cells, with clear decreases in DTPs surviving combination treatment at all time points when compared with control mimic-transfected cells (Figure 4A). In sotorasib-treated H358 cells, there were fewer surviving DTPs, and this fraction was further inhibited by transfection with mimics (Figure 4B).

To further investigate the impact of construct transfection in the context of continued osimertinib pressure, proliferation and viability were tracked for 6 weeks using a high-content imaging assay. PC9 cells transduced with mCherry were transfected with conmiR-15/107.4 or C81 control mimics (2 nM) and then treated with osimertinib. Monitoring confluency over time revealed that conmiR-15/107.4 significantly reduced the number of DTP cells compared with control-transfected cells at early time points (Figure 4C). The delay in proliferation was maintained throughout the observation period, although the response in each replicate well was heterogeneous (Figure 4D; additional wells in Supplementary Figure S2). From the comparison of individual wells, it is clear that despite the transfection method used resulting in >95% transfection efficiency [36], the response to osimertinib of cells in parallel conmiR-15/107.4-transfected wells ranged from complete inhibition of cell survival, through colony formation to full confluency by week 6 (Figure 4D and Supplementary Figure S3). This is in contrast to the effects of control-transfected cells, for which cell proliferation in the presence of osimertinib was more uniform, with almost all wells reaching confluency by 3 weeks of continued treatment (Figure 4C,D).

## 3. Discussion

In recent years, miRNA mimics have emerged as a promising therapeutic strategy due to their ability to regulate multiple oncogenic pathways simultaneously [37]. A notable example is the first-in-human phase 1 clinical trial of a miR-15/107–based mimic in PM, which demonstrated both objective clinical responses and good tolerability [32]. Building on these encouraging clinical findings, the present study extends the evaluation of this therapeutic approach to NSCLC models. Here, we report data showing the activity of a synthetic miR-15/107 consensus mimic in NSCLC and explore its potential to be used in combination with existing targeted therapies as an adjuvant strategy to enhance efficacy and delay resistance.

The conmiR-15/107 construct inhibited proliferation in both PC9 and H358 cells in a dose-dependent manner, and its efficacy was comparable to that of miR-15a, a native family member. This suggests that the consensus design effectively preserves the tumour-suppressive properties of the family, though it does not appear to confer substantially greater potency. Previous reports have described stronger activity relative to miR-16 [30], but such discrepancies may reflect differences in comparator choice, cellular context, or overlapping target repertoires. Importantly, inhibition of proliferation observed here is consistent with earlier studies of miR-15/107 mimics in adenocarcinoma models and aligns with the efficacy reported for other therapeutic miRNA mimics, including miR-34a and let-7 [38,39]. Together, these findings indicate that consensus-based constructs can match the performance of natural family members while potentially broadening applicability across tumour types by converging on shared oncogenic effectors.

The introduction of conmiR-15/107 constructs enhanced the activity of multiple targeted therapies in NSCLC models, encompassing both EGFR-mutant and KRAS-mutant cell lines. ConmiR-15/107 constructs increased the effectiveness of EGFR inhibition in PC9 cells, enhancing responses to both gefitinib and osimertinib. This effect may result from complementary inhibition of the ERK/MAPK pathway, whereby EGFR inhibitors attenuate upstream signalling, while conmiR-15/107 is predicted to suppress downstream effectors such as MEK1. These observations are consistent with prior reports. Fanini et al. [25] showed that a miR-16 mimic increased A549 sensitivity to erlotinib by targeting key ERK/MAPK components, including MAPK3 (ERK1), MAP2K1 (MEK1) and CRAF. Multiple previous studies have found that a variety of miRNA mimics targeting EGFR or the EGFR-signalling pathway can improve the efficacy of EGFR-TKIs, for example, gefitinib [40,41,42]. This sensitisation effect extended to gefitinib-resistant cell lines in many cases. More broadly, tumour-suppressive miRNAs such as miR-34a and let-7 have been paired with first- and third-generation EGFR-TKIs, yielding potentiated—and in some studies synergistic—inhibition of proliferation [29,30,43].

In KRAS^G12C^ mutant H358 cells, the conmiR-15/107 constructs enhanced responses to the KRAS^G12C^-specific inhibitors sotorasib and adagrasib. This indicates that the constructs may influence downstream pathways in a manner that enhances cellular responsiveness to KRAS blockade. Although previous studies have shown that miR-16 can target KRAS [44] and senstise lung adenocarcinoma cells to targeted therapies [25], to our knowledge, the effect of miR-15/107 family members on response to KRAS^G12C^ inhibitors has not been tested, suggesting that this may represent a novel therapeutic approach. This is supported by other RNA-based strategies targeting KRAS that have entered the clinic. For example, the iExoKRAS^G12D^ Phase I trial (NCT03608631) tested engineered exosomes loaded with KRAS^G12D^-specific siRNA in pancreatic cancer patients [45]. The therapy was well tolerated and showed molecular evidence of target engagement, including reduced KRAS signalling and increased CD8+ T-cell infiltration. Similarly, an ASCO-reported Phase I trial evaluated KRAS^G12D^ siRNA-loaded exosomes, also demonstrating safety and signs of disease stabilisation in advanced cases of pancreatic ductal adenocarcinoma [46].

There is also growing evidence that combining KRAS inhibition with MAPK pathway targeting can improve therapeutic efficacy [47,48]. Further support for RNA-based combination approaches comes from Xue et al., who showed that siRNA-mediated KRAS knockdown, when paired with a miR-34a mimic delivered via lung-targeting nanoparticles, produced marked tumour growth inhibition in NSCLC mouse models [49]. Additional support for RNA-based strategies targeting KRAS comes from the work of Acunzo et al., who designed artificial miRNAs (amiRs) that selectively target point-mutated KRAS^G12S^ and KRAS^G12D^ transcripts while sparing the wild-type allele [50]. Although their study focused on direct allele-specific silencing rather than pathway modulation, it reinforces the broader principle that engineered miRNA-based therapeutics can be harnessed to overcome the limitations of current targeted therapies in the treatment of KRAS-mutant NSCLC.

The addition of conmiR-15/107 constructs significantly suppressed DTP outgrowth under osimertinib pressure, a finding observed in both the 19-day assay and corroborated by 6-week live-cell imaging. Multiple factors may contribute to the early suppression of DTP emergence. Among them, the targeting of downstream effectors of the EGFR pathway—such as MEK1—by the miRNA constructs could contribute to the blockade achieved by EGFR inhibitors. Despite the encouraging early effects, DTPs eventually re-emerged, reflecting the short-lived activity of miRNA mimics [51]. Their efficacy is confined to the immediate post-transfection window, as dilution during cell division quickly reduces activity, limiting sustained suppression. The re-emergence of DTP populations and the variability in their delay were consistently observed. This variability reflects a key limitation of miRNA-based therapy—one shared with conventional targeted treatments—namely the inherent heterogeneity of DTPs [52,53]. Within otherwise sensitive populations, rare cells express higher levels of proteins that confer tolerance, influenced by stochastic fluctuations or epigenetic variability [54,55]. The abundance and state of these pre-DTP subpopulations at the time of drug exposure critically shape both the initial response and timing of regrowth.

Despite limited evidence on the role of the miR-15/107 family in DTPs, several other miRNAs have been directly implicated in persister cell biology. In EGFR-mutant NSCLC models, miR-147b was upregulated in osimertinib-tolerant cells and promoted a reversible tolerant state through TCA cycle disruption and pseudohypoxia [56], while miR-21-5p/3p drove tolerance by repressing ADSL and rewiring purine metabolism [57]; inhibition of either pathway restored drug sensitivity. Broader screening approaches also support a central role for miRNAs in tolerance. In a large-scale analysis of PC9-derived DTP cells, multiple miRNAs were found to be downregulated, among them miR-371-3p, which normally acts to suppress persister formation by targeting PRDX6 and reducing redox buffering [58]. The loss of tumour-suppressive miRNAs in DTPs highlights how changes in the miRNA landscape actively shape tolerance phenotypes.

The miR-15/107 family remains a promising therapeutic axis, yet our findings underscore that durable efficacy will require strategies capable of overcoming both delivery challenges and the heterogeneity of persister populations. Novel platforms such as Selective Organ Targeting (SORT) lipid nanoparticles [59,60] and tumour-targeted minicells, exemplified by the TARGOmiR trial [32], offer potential solutions. SORT LNPs incorporate charge-modifying components that enable organ-selective distribution, and encouragingly, preclinical studies have demonstrated their ability to deliver functional small RNAs, including siRNAs, to the lung [61,62]. However, these systems remain untested in humans, and important limitations persist, including a lack of tumour specificity, inefficient endosomal escape [63] and the risk of systemic toxicity [31,64]. To try and address these concerns, chemically modified miRNAs combined with targeted ligand conjugation [65] and endosomal escape agents have been developed [66]. For example, the DUPA-nigericin-miR-34a have demonstrated selective uptake by PSMA-positive cells and a corresponding decrease in proliferation in vitro and in vivo [66].

To move toward clinical application, future studies will need to evaluate the activity of conmiR-15/107 in vivo, where tumour complexity, immune interactions and delivery barriers will determine efficacy. Ultimately, rational combinations with targeted therapies and adaptive approaches that address DTP heterogeneity will be required to translate these encouraging findings into durable clinical benefit. In conclusion, conmiR-15/107 enhances the initial activity of EGFR and KRAS inhibitors and postpones the emergence of persister cells, demonstrating clear potential as an adjuvant strategy in NSCLC. However, the transient activity of miRNA mimics and the heterogeneous nature of persister populations remain significant hurdles. Overcoming these limitations through improved delivery technologies and strategic combination regimens will be critical to advancing consensus-based miRNA therapeutics toward clinical application.

## 4. Materials and Methods

### 4.1. Cell Culture

A549, BEAS-2B, PC9 and H358 cell lines were obtained from ATCC (American Type Culture Collection, Manassas, VA, USA). Cells were grown in RPMI 1640 medium with GlutaMax (Thermo Fisher Scientific, Hillsboro, OR, USA) supplemented with 10% fetal bovine serum (Moregate BioTech - Hamilton, New Zealand). All the cell lines were regularly tested for Mycoplasma (Mycoalert Mycoplasma Detection Kit, Lonza, Basel, Switzerland). The cell line identity was confirmed using STR profiling carried out by Cell Bank Australia (Sydney, Australia).

### 4.2. Transfections

Reverse transfections were performed in all experiments using miRNA mimics or siRNAs (outlined in Supplementary Table S1) and Lipofectamine RNAiMax (Thermo Fisher Scientific). All transfections were performed using the manufacturer’s recommendations for Lipofectamine RNAiMax (Thermo Fisher Scientific). The conmiR-15/107 constructs consisted of a consensus mimic as described previously [30]. The mimic of endogenous miRNA miR-15a consisted of the mature miRNA sequence and the passenger strand. RRM1 siRNA (targeting the M1 subunit of ribonucleotide reductase) was employed as a positive control for transfection efficiency, as its successful delivery is known to induce robust cell death [66,67]. C81, a previously described siRNA with no effect on cell proliferation and no known targets in the human genome [66,67], was employed as a negative control.

### 4.3. Cell Proliferation Assays

To determine the half-maximal inhibitory concentration (IC_50_) of candidate compounds, serial dilutions were prepared and applied to cells seeded in 96-well plates. Cells were reverse-transfected with miRNA mimics at the indicated concentrations, and after 24 h, treated with the indicated targeted therapy drug under the respective experimental conditions. Cells were then allowed to grow until controls reached confluency (96 h following transfection), at which point proliferation was assessed using a SYBR Green-based assay (Thermo Fisher Scientific), as previously described [66,67]. Non-linear regression analysis was used to determine an estimated IC_50_ value of the resulting proliferation curve.

### 4.4. RT-qPCR

To determine the expression of target mRNA, transfections were performed in 6-well plates with miRNA mimics (2 nM). After 24 h, cells were treated with the respective targeted therapy or vehicle control (DMSO). Following incubation (48 h), RNA was extracted using the PureLink™ RNA Mini Kit (Thermo Fisher Scientific) following the manufacturer’s instructions. RNA concentration and quality were determined by NanoPhotometer N60 (Implen Inc., Westlake Village, CA, USA). Isolated RNA (1000 ng) was used to make cDNA. qScript cDNA Synthesis Master Mix (Quantabio, Beverly, MA, USA) was used according to the manufacturer’s instructions. Thermal cycling conditions were as follows: annealing for 5 min at 25 °C; cDNA synthesis for 30 min at 42 °C; and denaturing for 5 min at 85 °C. The resulting cDNA was diluted (10 ng/µL), and RT-qPCR reactions were performed with TB Green^®^ Premix Ex Taq™ II (Tli RNaseH Plus, Takara Bio—San Jose, CA, USA) using 10 ng cDNA per reaction and forward and reverse primers (4 nM; Supplementary Table S2). A ROX reference dye (Takara) was included as an internal control. Ubiquitin C (UBC) was used as a reference gene for normalisation, with relative expression calculated using the 2^−ΔΔCT^ method [68].

### 4.5. Target Protein Expression

To assess target protein expression, cells were transfected with miRNA mimics (2 nM) in 10 cm dishes. After 24 h, the media was replaced, and following an additional 48 h, the cells were treated with the respective targeted therapy or vehicle control (DMSO). After a 4 h incubation, the media was removed and washed with ice-cold PBS. Proteins were extracted using 1× RIPA buffer (NaCl 150 mM, NP40 1% *v*/*v*, sodium deoxycholate 0.5% *v*/*v*, SDS 0.1% *v*/*v*, Tris (pH 7.4) 25 mM) supplemented with protease/phosphatase inhibitors (Roche, Basel, Switzerland). Protein concentration was determined using the Pierce BCA Protein Assay Kit (Thermo Fisher Scientific). Equal amounts of protein (50 µg) were mixed with 1× Laemmli buffer and 5% *v*/*v* 2-mercaptoethanol (Sigma-Aldrich, Burlington, MA, USA), then denatured at 98 °C for 5 min. Samples were separated on a Mini-PROTEAN TGX 10-well precast gel (4–20% gradient, Bio-Rad, Hercules, CA, USA) alongside Precision Plus Protein Dual Color Standards (Bio-Rad) and subsequently transferred to a nitrocellulose membrane (0.2 μm pore size) for antibody staining. The following primary antibodies were used at 1:1000 dilution in blocking buffer (5% trim milk in TBS-Tween [0.1% Tween]): anti-p44/42 MAPK (ERK1/2) (#9102 Cell Signalling Technology, Danvers, MA, USA) and anti-phospho-p44/42 MAPK (pERK1/2) (#9101 Cell Signalling Technology). The secondary antibody (IRDye^®^ 680RD, Goat anti-Rabbit, LI-COR, Lincoln, NE, USA) was used in a 1:20,000 dilution in blocking buffer. Western blots were imaged using the LI-COR ODESSEY CLx imaging system (Waltham, MA, USA). The software package Image Studio Software Version 6.0 (LI-CoR) was used to analyse the images.

### 4.6. Long-Term Proliferation Assays

To investigate the impact of conmiR mimics on drug tolerance, proliferation assays were performed in either 6- or 96-well plates. PC9 or H358 cells were reverse-transfected in 6-well plates with miRNA mimics (2 nM) and 1 day later treated with the respective targeted therapy. Drug treatments were renewed every 4 to 5 days, and cells were maintained under these conditions for 3, 7 or 19 days to generate DTPs [34]. At each timepoint, wells were photographed and stained with crystal violet (Sigma-Aldrich) to assess cell proliferation and confluence. Longer-term assays were performed to assess the proliferation of transfected cells over six weeks. PC9 cells stably expressing pSin-mCherry-PuroR [35] were transfected in Greiner µClear glass-bottom 96-well plates. Twenty-four hours post-transfection, cells were treated with 2 µM osimertinib, with drug-containing media refreshed weekly, 1 day prior to imaging. Plates were imaged weekly using the Opera Phenix High Content Screening System (Perkin Elmer, Waltham, MA, USA). Brightfield and mCherry fluorescence images were acquired with a 5× air objective (NA 0.16, non-confocal), using the following settings: Brightfield (10 ms exposure, 5% laser power and 90.0 µm focal height) and mCherry (250 ms exposure, 100% laser power, −88.0 µm focal height). Image acquisition and pre-processing were carried out using Harmony Software (v5.1, Revvity, Waltham, MA, USA), and data were subsequently analysed in Signals Image Artist (v1.0, Revvity).

### 4.7. Statistical Analysis

Proliferation assays and RT-qPCR were performed with an average of three biological replicates. The statistical difference for the IC_50_ proliferation assays was calculated between the transfection conditions at each concentration of the targeted therapy using a two-tailed *t*-test. Statistical differences between RT-qPCR experimental groups and the confluency estimates in long-term proliferation assays were evaluated using an unpaired, non-parametric Mann–Whitney test in GraphPad Prism (version 10). A *p*-value < 0.05 was considered significant in these experiments.

## Data Availability

The original contributions presented in this study are included in the article/Supplementary Materials. Further inquiries can be directed to the corresponding author.

## Supplementary Materials

The following supporting information can be downloaded at: https://www.mdpi.com/article/10.3390/ijms27062701/s1. References [69,70] are cited in the Supplementary Materials.
