# Supplementary material for: MicroRNA Mimics Based on the miR-15/107 Consensus Sequence Sensitise NSCLC Cells to Targeted Therapy
Source: Int J Mol Sci. 2026 Mar 16;27(6):2701. doi: 10.3390/ijms27062701 (PMC13026349; doi:10.3390/ijms27062701)
Supplement: Supplementary file 1 [file ijms-27-02701-s001.zip › ijms-4138941-supplementary.pdf]

Supplementary Material

Supplementary Table S1: siRNA and microRNA mimic sequences

**Name**	Antisense **(5'-3')**	Sense **(5'-3')**
RRM1	AAGAUCUGCUUAUUCAGUAACUGGG	mCmCmCmAmGmUUACUGAAUAAGCA mGmAmUmCmUmU
C81	AAGCAACUUGGUAAGACUCGUGUGG	mCmCmAmCmAmCGAGUCUUACCAAG mUmUmGmCmUmU
conmiR-15/107.2	UAGCAGCACAUAAUGUUUGCGGA	mUmCmCmGCAAACCAUUAUGUGCUmG mCmUmA
conmiR-15/107.4	UAGCAGCACAGUAUGGUUUGCG	mCmGmCmAAACCAUACUGUGCUmGmC mUmA
miR-15a	UAGCAGCACAUAAUGGUUUGUG	mCmAmCmAAACCAUUAUGUGCUmGm CmUmA

'm' represents 2'O-methyl modifications

Supplementary Table S2: Primer sequences used for RT-qPCR.

Primer target	**Forward (5'-3')**	**Reverse (5'-3')**
UBC	GCAAAGATCCAAGATAAGGAA	GGACCAAGTGCAGAGTGGAC
RRM1	GGCAAACTCACTAGTATGCACTTC	AAATAATACATCCCAGTCTTCAAACC
BCL2	AAAAATACAACATCACAGAGGAAGT	GTTTCCCCCTTGGCATGAGA
MAP2K1	CAATGGCGGTGTGGTGTTC	GATTGCGGGTTTGATCTCCAG
BRCA1	AGAAACCACCAAGGTCCAAAG	GGGCCCATAGCAACAGATTT
FGFR1	AATGAGTACGGCAGCATCAAC	ACCTCGATGTGCTTTAGCCAC
IGF1R	ATGCTGACCTCTGTTACCTCT	GGCTTATTCCCCACAATGTAGTT
CCND1	GCTGCGAAGTGGAAACCAT	CCTCCTTCTGCACACATTTGAA

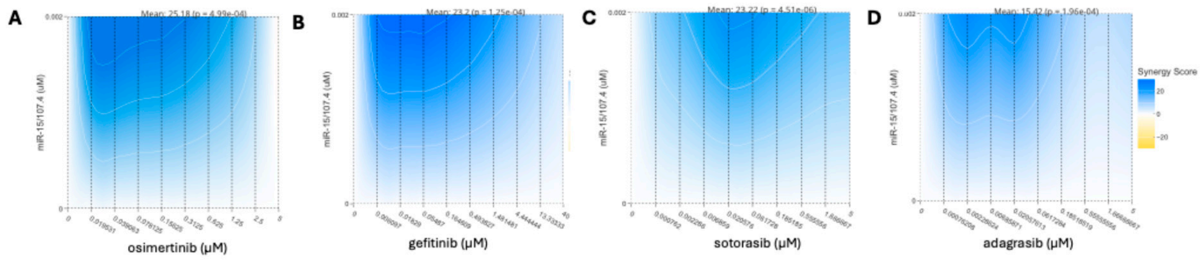

Supplementary Figure S1: Synergy analysis of conmiR-15/107.4 combined with targeted therapy. 2D synergy contour plots of conmiR-15/107.4 with **(A)** osimertinib or **(B)** gefitinib in PC9 cells, or with **(C)** sotorasib or **(D)** adagrasib in H358 cells. Synergy analysis was conducted using the SynergyFinder Plus software package [1] with the Zero Interaction Potency (ZIP) model [2] to statistically evaluate whether the observed effects in the combination assays were synergistic, additive, or antagonistic. The ZIP model was selected for the analysis, as it accounts for both the potency and shape of dose-response curves when assessing drug interactions. The 2D contour plot illustrates the interaction landscape between targeted therapy and the conmiR-15/107.4 construct. Each point represents a unique dose combination, with the colour gradient indicating the ZIP synergy score. Blue regions denote synergistic interactions, white indicates additive effects, and yellow represents antagonism.

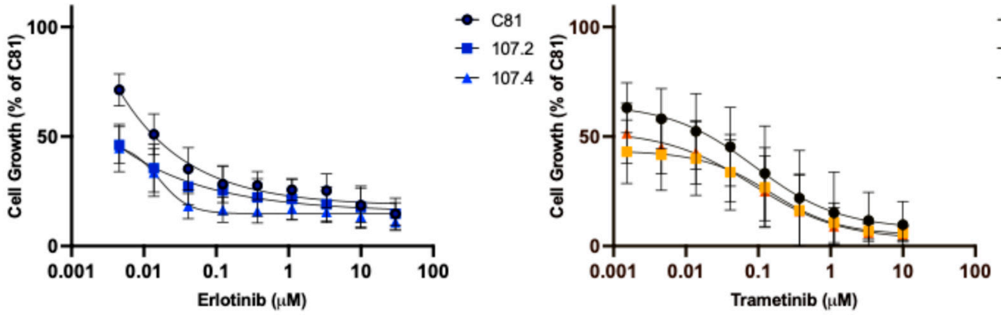

Supplementary Figure S2: conmiR-15/107 constructs in combination with targeted therapy efficacy. Dose-response curve showing cell growth of PC9 **(A)** and A549 **(B)** cells transfected with C81 (negative control), conmiR-15/107.2, or conmiR-15/107.4, and treated with increasing concentrations of erlotinib **(A)** and trametinib **(B)**. Data represent mean ± SD of biological replicates, normalized to C81 control.

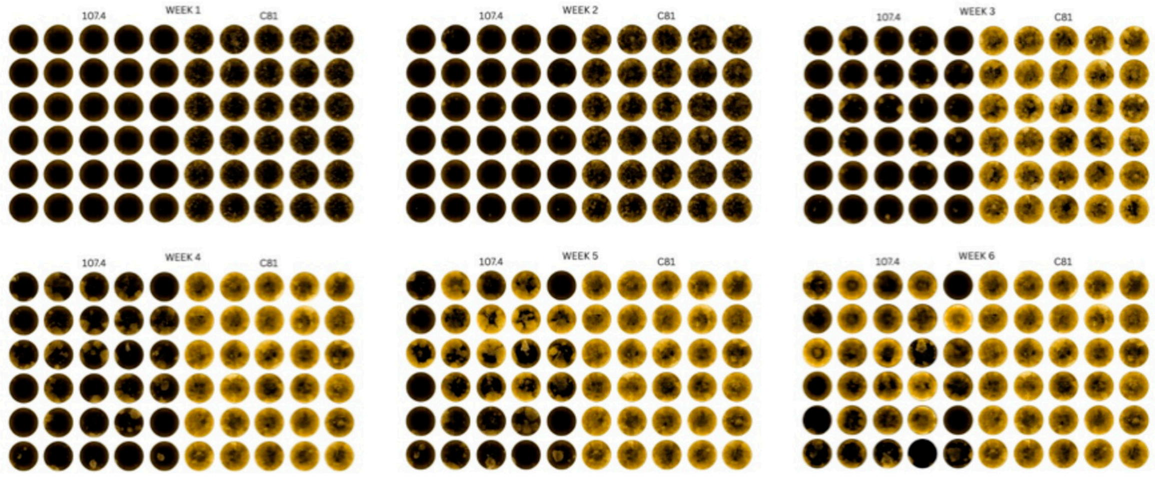

Supplementary Figure S3: High-content imaging of NSCLC cells transfected with control (C81) or miR-107.4 constructs under targeted treatment, over a period of 6 weeks. Representative wells (n=30) from three independent plates per condition (180 wells/condition) are shown. A random grid generator was used to select which 30 wells per condition to follow across the three replicate plates. These wells were then monitored over a six-week period. Each panel corresponds to the time point indicated, with images acquired using the Phenix high-content imaging system.

References:

1. Zheng, S., et al., *SynergyFinder Plus: Toward Better Interpretation and Annotation of Drug Combination Screening Datasets.* Genomics Proteomics Bioinformatics, 2022. **20**(3): p. 587-596.
2. Yadav, B., et al., *Searching for Drug Synergy in Complex Dose–Response Landscapes Using an Interaction Potency Model.* Computational and Structural Biotechnology Journal, 2015. **13**: p. 504-513.
